# Effect of Yijinjing combined with elastic band exercise on muscle mass and function in middle-aged and elderly patients with prediabetes: A randomized controlled trial

**DOI:** 10.3389/fmed.2022.990100

**Published:** 2022-11-03

**Authors:** Yunda Huang, Junhua Han, Qing Gu, Yanwei Cai, Jingyuan Li, Shasha Wang, Suijun Wang, Ru Wang, Xiangyun Liu

**Affiliations:** ^1^Shanghai University of Sport, Shanghai Frontiers Science Research Base of Exercise and Metabolic Health, Shanghai, China; ^2^Yinhang Community Health Center, Shanghai, China; ^3^Department of Endocrinology, Shidong Hospital, Shidong Hospital Affiliated to University of Shanghai for Science and Technology, Shanghai, China

**Keywords:** Qigong Yijinjing, elastic band exercise, physical performance, lean mass, glycemic control, prediabetes

## Abstract

**Introduction:**

This study investigated the effect of Yijinjing combined with elastic band exercise on muscle mass and muscle function in patients with prediabetes.

**Methods:**

This study was a randomized controlled trial designed in parallel (Chinese Clinical Trial Registry: ChiCTR2000039049). Participants with prediabetes (*n* = 47) were randomly divided into control (*n* = 21, 63.5 ± 4.7 years,16 females) and exercise (*n* = 26, 62.0 ± 5.0 years, 20 females) groups. The former maintained their original lifestyle, and the latter received Yijinjing combined with elastic band exercise five times a week for 6 months. All the outcome measures were assessed immediately at baseline, after 3- and 6-month intervention.

**Results:**

After 6-month of the exercise, the body weight, body mass index, leg fat mass, gynoid fat mass, and total body fat mass in exercise group were significantly decreased compared with those at baseline (*p* < 0.05). Compared with those at baseline, total lean mass decreased at 3 and 6 months in both groups. The total muscle mass loss in the exercise group was always less than that in control group at all time periods, but the difference was not statistically significant. Handgrip strength, gait speed, reaction time, leg power, eye-closed and single-legged standing, and sit-and-reach were significantly improved for the exercise group at 3 and 6 months (*p* < 0.05). Gait speed and reaction time between both groups at 3 and 6 months were significant different (*p* < 0.05), and leg power at 6 months (*p* < 0.05). Compared with baseline, the reaction time of control group at six months was significantly improved (*p* < 0.05), and no other significant changes were observed. Compared with those at baseline, fasting plasma glucose, 2-h post-meal plasma glucose, fasting insulin, total cholesterol, and insulin resistance index in exercise group gradually decreased, and growth hormone was gradually increased with significance at 6 months (*p* < 0.05). 25-hydroxyvitamin D gradually and significantly increased in both groups at 3 and 6 months (*p* < 0.05). But two groups’ testosterone levels weren’t significant change.

**Conclusion:**

Yijinjing combined with elastic band exercise can substantially reduce the body weight and body fat content of middle-aged and elderly patients with Prediabetes, improve muscle function and growth hormone secretion, and delay muscle mass reduction and diabetes development.

**Clinical trial registration:**

[http://www.chictr.org.cn/showproj.aspx?proj=62753], identifier [ChiCTR2000039049].

## Introduction

The population of people with diabetes mellitus (DM) and prediabetes mellitus (PDM) is globally increasing due to lifestyle changes and prolonged life expectancy (1). The prevalence of PDM (early-stage diabetes) is as high as 35.70% (1) and that of the elderly ranged from 45 to 47% (2) in China. Sarcopenia is an age-related skeletal muscle atrophy syndrome characterized by muscle mass loss, decreased muscle strength, and declined muscle function (3). A recent meta-analysis revealed that DM was associated with an increased risk of sarcopenia (4). Older adults with DM have a threefold increased risk of sarcopenia and a 50–80% increased risk of muscle dysfunction compared with those without (5, 6). Muscle mass loss reducing insulin target tissues and glucose metabolism leads to insulin resistance (7). Kalyani et al. found that Insulin resistance causes the reduced stimulation of protein synthesis pathways and the enhanced activation of protein degradation pathways that could finally result in muscle loss (8). Low-grade inflammation, mitochondrial dysfunction, and hormone level changes caused by insulin resistance further interfere with muscle cell function and even their death, thereby exacerbating muscle atrophy. Muscle mass loss, decreased muscle strength, and deteriorated muscle function further inhibit physical activity and aggravate the PDM and DM (9). Therefore, the prevention and treatment of early-stage diabetes (PDM) and its myopathy are significant.

Sarcopenia belongs to the muscle impotence disease in Traditional Chinese Medicine (TCM). TCM interprets sarcopenia as the result of overtendon contracture. Like Tai Chi, Qigong Yijinjing is a traditional Chinese mind-body exercise (10), and has a variety of movements and is safe and easy to learn. Its effect of “weak tendons are easy to strengthen, strong tendons can be stronger” is globally recognized. Cheng et al. found that Yijinjing can improve skeletal muscle contraction function by pulling muscle groups, fascia, tendons, ligaments, and other connective tissues in various parts of the body to promote qi (Qi, ancient Chinese cognition of the body, is similar to blood and sustains life.) and blood circulation and activate stiff muscles and joints (11). Elastic band exercise, one of the common forms of resistance exercise, has the advantages of high safety, simplicity and portability, small site requirements, reasonable price, and suitability for most patients with PDM. Elastic bands similarly improve skeletal muscle strength and life quality and even have better effects on functional movement ability than traditional resistance exercise (12). The study by Chen et al. showed that elastic band exercise could substantially improve muscle strength, dynamic balance, and body function in elderly people with DM and knee osteoarthritis (13). Furthermore, Colleluori et al. found that compared with aerobic or resistance exercise alone, aerobic exercise combined with resistance training could reduce body fat in obese patients and promote muscle protein synthesis, thereby protecting their muscle mass (14). Thus, the combined exercises have a better effect on muscle mass and function.

Aerobics combined with resistance exercise has become the basic treatment for DM. The American College of Sports Medicine (ACSM)’s guideline for physical activity in the middle-aged and elderly indicates that aerobic combined with resistance exercise is the best exercise method for DM, and recommends that older adults with type 2 diabetes mellitus (T2DM) get at least 150 min of moderate-intensity aerobic exercise per week, resistance exercise three times a week, and flexibility exercise no less than two times a week (15). This can help the patients to control blood sugar, improve physical function, and prevent falls. Yijinjing is not only moderate aerobic exercise but also has the function of stretching flexibility. Yijinjing combined with elastic band exercise conforms to the exercise guidelines recommended by ACSM. Hence, it was selected as the main intervention measure.

Very few studies have been conducted on muscle mass and function in people with PDM, and the effects of Yijinjing combined with resistance exercise in this population remain unclear. Therefore, this study explored the effect of Yijinjing combined with elastic band exercise on glycolipid metabolism, body composition, and physical performance of the PDM population, in order to find a safe and effective exercise and help them delay muscle atrophy, motor ability decline, and diabetes development. The combined exercise was hypothesized to improve blood glucose control, prevent muscle atrophy, and enhance muscle function in patients with PDM.

## Methods

### Participants

Participants with PDM were recruited from Shanghai Shidong Hospital in China from December 2020 to April 2021. Inclusion criteria were as follows: had met the diagnostic criteria for PDM (fasting plasma glucose < 7.8 mmol/L and/or 7.8 ≤ OGTT2h blood glucose < 11.1mmol/L) (16); not exercising regularly for the last 6 months; no smoking or drinking in the past 1 year; voluntarily participated and signed the informed consent; aged 45–75 years; and has normal cardiopulmonary function and ability to perform fitness tests. Exclusion criteria were as follows: had a recent injury or started taking hypoglycemic drugs; participating in other clinical trials; has high-risk or special disease (cardiovascular disease, cancer, and so on); and has a metal stent or pacemaker placed inside the body.

### Sample size calculation

On the basis of a previous randomized controlled trial examining the effect of resistance exercise on muscle mass and physical performance in elderly patients with sarcopenia (17), the sample size was designed to identify intergroup differences in 1,000 g of lean body mass after 26 weeks of different interventions with assumed standard deviation of 1,100 g, α level of 0.05, and test efficacy of 80%. The sample size was estimated to be at least 21 participants per group. With an expected shedding rate of 20%, the minimum number of people to be enrolled was set at 52.

### Study design

This study was a randomized controlled trial designed in parallel. The participants were randomly assigned to control group (CG) and exercise group (EG) in a 1:1 ratio by generating random numbers using SPSS software. Randomization and assignments were accomplished by persons not involved in the participant recruitment. Each index was measured at 1 week baseline, 3 months, and 6 months. This study protocol was registered in the Chinese Clinical Trial Registry (ChiCTR2000039049) and approved by the Ethics Committee of Scientific Research of Shanghai University of Sport (Ethics batch number: 102772021RT066). All study participants signed informed consent forms.

### Intervention program

The CG maintained their original living and eating habits without any exercise intervention. According to ACSM’s Exercise Guidelines for T2DM patients (15), the EG underwent Yijinjing combined with elastic band exercise 5 times a week (Monday to Friday, 8:00 a.m. to 9:00 a.m.) for 6 months (Table 1). Twelve types of Yijinjing were included as follows: Wei Tuo Presenting the Pestle 1, Wei Tuo Presenting the Pestle 2, Wei Tuo Presenting the Pestle 3, Plucking a Star and Exchanging a Star Cluster, Pulling Nine Cows by Their Tails, Displaying Paw-Style Palms like a White Crane Spreading Its Wings, Nine Ghosts Drawing Swords, Three Plates Falling on the Floor, Black Dragon Displaying Its Claws, Tiger Springing on Its Prey, Bowing Down in Salutation, and Swinging the Tail. Five types of elastic band exercise were included as follows: Straight-arm chest expansion, Forward lunge biceps bend (left and right), Knee resistance squats, standing waist extension (left and right), Full-body resistance advanced squats. Males use 35-pound Jingyuan Jingyuan Li-Ning elastic band (Blue, 1,500 mm × 150 mm × 0.5 mm), females use 25-pound Li-Ning elastic band (Blue, 1,500 mm × 150 mm × 0.4 mm). During the exercise, professional coaches and medical staff supervised and guided each patient to ensure the safety and correctness of movements and stimulate the enthusiasm to participate in the exercise. Participants used Polar Team 2 monitors to monitor heart rate in real time during exercise. Determine whether the heart rate during exercise is within the range of Target Heart Rate (THR) of moderate intensity to assess exercise intensity (THR = (Maximum Heart Rate-Resting Heart Rate) × Exercise Intensity + Resting Heart Rate, Maximum Heart Rate = 207 − 0.7 × age, Expected Exercise Intensity is 60–70%). If the intensity of exercise falls below moderate intensity, then the patient is encouraged to adjust the posture of exercise, the degree of stretching, or the grip distance of the elastic band to increase the intensity.

**TABLE 1:** Exercise intervention program.

	Warm up	Aerobic exercise	Resistance exercise	Cool down
**Phase 1**	**Activity:** Neck flexion and rotation, chest expansion, waist rotation, air Squat, ankle activity **Duration:** 5min **Reps:** 4 × 8 beats	**Activity:** Twelve types of Yijinjing exercise **Duration:** 28 min **Reps:** twice **Intensity:** THR **Interval:** 60 s	**Activity:** Five types of elastic band exercise **Duration:** 12 min **Reps:** twice **Intensity:** THR **Interval:** 60s	**Activity:** stretch-neck/shoulder/chest/waist/arm/ leg/ankle **Duration:** 5min **Reps:** 4 × 8 beats
**Phase 2**	**Activity:** As in phase 1 **Duration:** 5 min	**Activity and Intensity:** As in phase 1 **Duration:** 39 min **Reps:** 3 times **Interval:** 30 s	**Activity and Intensity:** As in phase 1 **Duration:** 18 min **Reps:** 3 times **Interval:** 30 s	As in phase 1
**Phase 3**	**Activity:** As in phase 2 **Duration:** 5 min	**Activity, Duration, Reps, Intensity, and Interval:** As in phase 2	**Activity**, **Intensity and Interval:** As in phase 2 **Duration:** 24 min **Reps:** 4 times	As in phase 2

THR, target heart rate; s, second; min, minute; Reps, repetitions.

### Clinical measurements

Body composition (muscle and fat content in various parts of the body) was measured by dual energy X-ray absorptiometry (DXA, GE Medical System, Lunar Prodigy, software version: 12.2) (18), Skeletal muscle mass index was calculated by dividing muscle mass (kg) by height squared (m^2^). Prior to the test, the participant was in a fasted state. During the test, the participants removed all metal items from the body, took off the coat, put on the test suit, lay flat in the specified area of the scanning bed, and kept an upright and supine position for DXA scanning. Body mass index (BMI) was weight divided by height squared (m^2^).

Waist and hip circumference were measured with a soft tape ruler, and the former divided by the latter was the waist-to-hip ratio. Waist circumference measurement method: the soft tape ruler was horizontally circled 0.5–1 cm above the navel (the thickest waist of obese people), and the measurement value at the end of expiratory was taken. Hip circumference measurement method: the soft tape ruler was horizontally circled around the most protrusion of gluteus maximus muscle, and the value intersecting “0” on the tape ruler was the measured value.

Handgrip strength was measured using a grip strength tester (Takei T.K.K. 5401, Niigata, Japan). The participants stood in an upright position with their hands holding the grip strength tester, extended their arms for 30°, palm inward, and held the internal and external grips with all their strength to keep their upper limbs fixed. The left and right hands were measured twice with an interval of 1 min for each measurement, and the maximum value of the left and right hands was recorded. Finally, the average value of the maximum value of the left and right hands was taken (19).

Gait speed was measured using the 4-m walk test. Four areas at the starting point, 1 m, 5 m, and the end point were marked along a 6-m straight line. The 4m gait speed was calculated as 4 m divided by walking time. The participants were asked to walk in a natural manner (19).

Body reaction time was measured using a reaction time meter (Takei T.K.K. 5401, Niigata, Japan). The participants stood on the sensor pad, knees slightly bent, and looked at the signal from the teleprompter. After the tester pressed the start button, the cue flashed red for 3–6 s. The participants jumped vertically away from the sensor pad as soon as possible. The tester then reset the test and waited for the next signal. Each participant completed five responses. The tester recorded the five times when the signal appeared until the foot completely left the induction pad and calculated the average in seconds (before the formal test, 5 attempts).

Leg power tester (Takei T.K.K. 5401, Niigata, Japan) was used to test the lower limb power. The participant was seated on the instrument stool with cushions back-to-back, seat belt fastened, feet fixed to the pedals, and hands holding both arm-rests. During the test, the participants pedaled as hard as they could for four times with an interval of 30 s for the best result.

Sit-And-Reach. Flexibility was measured using the automatic seat flexion tester (Takei T.K.K. 5401, Niigata, Japan). The participants sat on the mat with legs straight forward, heels together and toes naturally separated, and stepped on the baffle of the tester. During the test, both hands were together, palms down, flat extension forward, upper body forward bending. The participants pushed the cursor forward smoothly with the tips of middle fingers of both hands until they could not move forward. The test was performed twice, and the best result was selected.

Eye-closed and single-legged standing. Balancing ability was tested using the closed eye one-leg standing tester (Takei T.K.K. 5401, Niigata, Japan). During the test, the participants stood on the sensor board with their customary supporting feet, hands akimbo. Upon hearing the beep, the participants would raise their unsupported foot and then close their eyes. The tester recorded how long they maintained their balance. The end of the test was indicated by the landing of the participant’s unsupported foot or the instrument making another “drip” sound. The left and right feet were measured twice, and the best result was obtained.

Blood sample collection: Fasting venous blood was extracted from the anterior cubital vein of the participants who were in a fasted state, placed in the refrigerator at 4°C, and centrifuged at 3,500 r/min for 5 min to collect the supernatant, which was then put into a sterilized Eppendorf (EP) tube and stored in the refrigerator at −80°C for testing. Glycolipid metabolism and hormone markers were measured using an automatic biochemical analyzer (Thermo Scientific™ Indiko Plus). Blood glucose-related indicators were as follows: fasting plasma glucose (FPG), 2-h post-meal blood glucose (2hPG), fasting insulin (FINS), postprandial serum insulin (PSI). Lipid-related indicators were as follows: total cholesterol (TC), triglyceride (TG), high density lipoprotein cholesterol (HDL-C), low density lipoprotein cholesterol (LDL-C), and glycosylated hemoglobin% (HbA1c%). The formula for homeostasis model assessment (20) was as follows: “insulin resistance index (HOMA-IR) = fasting plasma glucose (mmol/L) × fasting insulin (mU/L)/22.5” and insulin action index(IAI) is invert of HOMA-IR.” Hormone indicators were as follows: testosterone (TTE), growth hormone (GH), and 25-hydroxyvitamin D (VD).

### Statistical analysis

Continuous variables were examined for normality by Shapiro-Wilk test. Values were expressed as mean ± standard deviation (95% confidence interval) or median (25%, 75%) for non-normally distributed data. Differences between groups were determined by independent sample T-test or Wilcoxon Mann–Whitney test for normally distributed data. Categorical data were compared using Chi-square test or Fisher’s exact test. The effect of Yijinjing combined with resistance exercise on blood glucose control, body composition, and physical performance were tested using 3 × 2 repeated measurement ANOVA. Time differences (baseline, after 3- and 6-month intervention) were considered as intra-subject, and the presence or absence of exercise intervention was considered as between-subject factor. A repeated measurement ANOVA was used to compare data from the same group at the three time points, and Bonferroni test was applied for *post-hoc* analysis. SPSS 21.0 statistical software was used for data processing, and significance level was defined as *p* < 0.05.

## Results

### Baseline characteristics of participants

**FIGURE 1:**
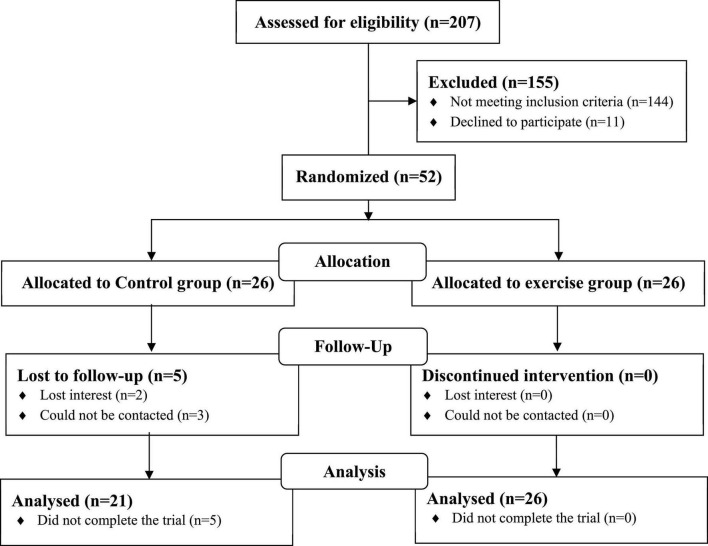
Participant flow diagram.

The flow diagram of participants was shown in Figure 1. Among the 207 participants who participated in routine blood tests, 52 met the inclusion criteria, agreed to participate, and signed the informed consent. Five participants in the CG dropped out of the experiment due to loss of interest (*n* = 2) or loss of contact (*n* = 3). Finally, 47 participants (36 women, 11 men) completed the 6-month trial (10% shedding rate) and were included in the study analysis. The average age of 21 participants (16 females and 5 males) in the CG and 26 participants (20 females and 6 males) in the EG was 63.5 ± 4.7 and 62.0 ± 5.0 years, respectively. Prior to the start of the intervention, both groups were comparable in terms of age, gender, lean mass, handgrip strength, gait speed, FPG, 2hPG, and so on. No significant group differences in baseline characteristics were found (all *p* > 0.05) (Table 2). Except for muscle soreness during the exercise, no exercise-related adverse events were recorded.

**TABLE 2:** Baseline characteristics of the participant in the study.

Variable	Control (*n* = 21)	Exercise (*n* = 26)	*P*-value
**Demographics and anthropometrics measurements**			
Age (year)	63.5 ± 4.7 (61.3–65.6)	62.0 ± 5.0 (60.0–64.0)	0.294
Gender (female/male), n/n	16/5	20/6	1.000
Weight (kg)	63.8 ± 10.3 (59.1–68.5)	64.7 ± 9.8 (60.7–68.6)	0.776
Body mass index (kg/m^2^)	23.9 ± 2.7 (22.7–25.1)	24.7 ± 3.0 (23.4–25.9)	0.380
Waist-to-hip ratio	0.90 ± 0.05 (0.88–0.92)	0.90 ± 0.05 (0.88–0.92)	0.869
Skeletal muscle index (kg/m^2^)	14.7 (13.6,16.0)	14.8 (14.0,16.3)	0.966
Total lean mass (kg)	38.1 (35.7,46.0)	38.0 (34.1,43.8)	0.781
Body fat rate (%)	32.8 (30.8,37.1)	36.9 (28.8,40.0)	0.113
**Physical performance**			
Handgrip strength (kg)	22.8 (19.9,31.7)	22.6 (21.4,27.7)	0.864
Gait speed (m/s)	1.14 ± 1.17 (1.06–1.21)	1.13 ± 0.13 (1.07–1.18)	0.855
Reaction time (s)	0.460 (0.386,0.517)	0.427 (0.401,0.427)	0.473
Leg power (W)	248.0 (168.0,301.5)	199.5 (162.0,288.0)	0.435
Eye-closed and single-			
legged standing (s)	6.0 (5.0,10.0)	5.0 (3.0,10.0)	0.250
Sit-And-Reach (cm)	5.4 ± 7.9 (1.7–9.0)	3.7 ± 9.8 (−0.3 to 7.7)	0.538
**Glycolipid metabolism**			
FPG (mmol/L)	5.85 (5.37,6.41)	6.17 (5.65,6.44)	0.181
2hPG (mmol/L)	9.48 (8.45,10.81)	9.47 (8.26,10.85)	0.740
HbA1c (%)	5.80 (5.65,6.10)	5.90 (5.50,6.13)	0.813
Fasting insulin (μU/mL)	9.30 (5.90,12.53)	9.40 (5.60,15.38)	0.872
Postprandial insulin (μU/mL)	73.70 (40.35,118.60)	46.70 (32.20,94.15)	0.171
Total cholesterol (mmol/L)	5.51 (5.11,5.85)	5.88 (4.68,6.72)	0.171
Triglyceride (mmol/L)	1.50 (1.26,2.27)	1.36 (0.99,1.90)	0.289
HDL−C (mmol/L)	1.38 (1.24,1.58)	1.53 (1.31,1.72)	0.104
LDL−C (mmol/L)	3.63 (3.37,3.80)	3.80 (2.98,4.45)	0.261
25−Hydroxyvitamin D (ng/ml)	16.80 (15.21,22.83)	16.57 (13.67,19.37)	0.404
Testosterone (ng/dL)	29.14 (14.68,163.09)	21.28 (14.54,78.69)	0.645
Growth hormone (ng/ml)	1.075 (0.634,1.293)	1.135 (0.630,1.346)	0.441
HOMA2−IR	2.501 (1.474,3.368)	2.571 (1.454,3.992)	0.669
IAI	0.400 (0.297,0.680)	0.389 (0.251,0.688)	0.669

Values are presented as means ± SD (95% confidence interval) or median (25%,75%) (if data are not normally distributed). FPG, fasting plasma glucose; 2hPG, 2-hour postprandial glucose; HbA1c%, glycosylated hemoglobin%; HDL-C, high density lipoprotein cholesterol; LDL-C, low density lipoprotein cholesterol. *p*-values represent baseline between-group comparisons.

### Body composition testing results

**FIGURE 2:**
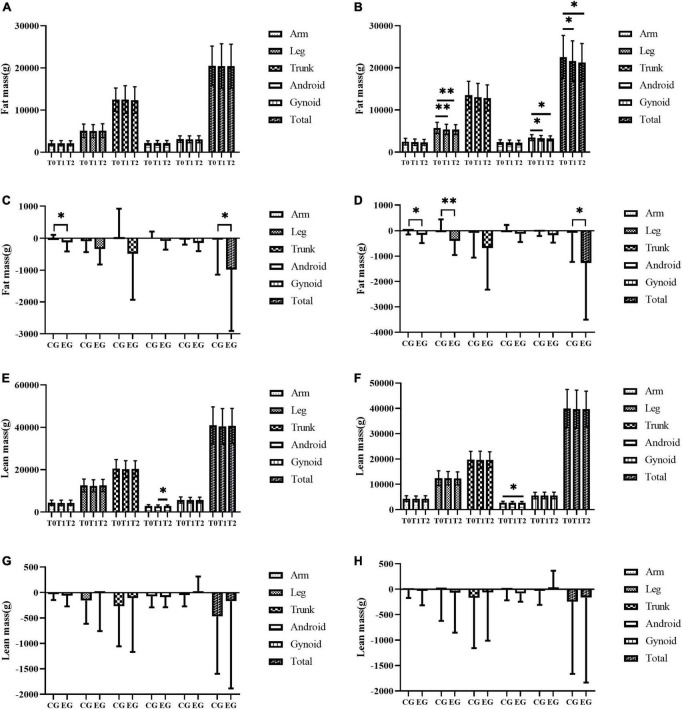
The effect of exercise on fat content and lean mass in all parts of the body. **(A–H)** The fat mass of all parts of the body in the control group **(A)**, the fat mass of all parts of the body in the exercise group **(B)**, comparison of difference values between three months and baseline in different groups on fat mass in all parts of the body **(C)**, comparison of difference values between 6 months and baseline in different groups on fat mass in all parts of the body **(D)**, the lean mass of all parts of the body in the control group **(E)**, the lean mass of all parts of the body in the exercise group **(F)**, comparison of difference values between three months and baseline in different groups on lean mass in all parts of the body **(G)**, comparison of difference values between six months and baseline in different groups on lean mass in all parts of the body **(H)**. T0, T1, and T2 represent baseline, 3 months, and 6 months respectively. CG and EG represent control group and exercise group respectively. * represents *p* < 0.05 and ** represents *p* < 0.01.

Body weight, BMI, leg fat mass, gynoid fat mass, and total body fat mass showed group and time interactions after 6 months of Yijinjing combined elastic band exercise, with the time effect being statistically different (Table 3). Compared with those at baseline, body weight, BMI, leg fat mass, gynoid fat mass, and total body fat mass were significantly decreased in the EG at 3 and 6 months (*p* < 0.05) (Table 3 and Figure 2B). No significant differences in all body composition indexes were observed in the CG (*p* > 0.05) (Table 3 and Figure 2A). The difference values of arm fat mass and total body fat mass between 3 months and baseline values were significant between the two groups (*p* < 0.05) (Figure 2C). In term of body weight, BMI, arm fat mass, leg fat mass, and total body fat mass, the difference values between 6 months and baseline values were significant between the two groups (*p* < 0.05) (Table 3 and Figure 2D). Compared with those at baseline, total lean mass decreased at 3 and 6 months in both groups. Android lean mass was significantly different between 3 and 6 months for the CG and was significantly lower at 6 months than that at baseline for the EG (*p* < 0.05). No significant change in muscle mass in the remaining parts was observed for the two groups (Figures 2E,F). The total muscle mass loss in the EG was always less than that in the CG at all time periods, but the difference was not statistically significant (Figures 2G,H). No significant changes were observed for waist-to-hip ratio, body fat%, and skeletal muscle mass index in the both groups. The EG showed a gradual downward trend of body fat%, but the change was not significant (Table 3).

**TABLE 3:** Effect of Yijinjing combined with resistance exercise on body composition in the study’s participant.

Body composition	Control (*n* = 21)	Difference from baseline	Exercise (*n* = 26)	Difference from baseline	*P*-value	*P*-value	*P*-value
					Group−time interaction	Time	Group
**Weight (kg)**					**0.025**	**0.002**	0.958
Baseline	63.8 ± 10.3 (59.1–68.5)		64.7 ± 9.8 (60.7–68.6)				0.776
three months	63.3 ± 10.9 (58.3–68.3)	−0.6 ± 1.6 (−1.3 to 0.2)	63.6 ± 9.9 (59.6–67.6)**a**	−1.1 ± 1.4 (−1.7 to −0.6)			0.927
six months	63.6 ± 11.2 (58.5–68.7)	−0.2 ± 2.2 (−1.2 to −0.8)	63.0 ± 9.4 (59.2–66.8)**b**	−1.7 ± 1.9 (−2.5 to −0.9)**e**			0.831
**Body mass index (kg/m2)**					**0.031**	**0.003**	0.549
Baseline	23.9 ± 2.7 (22.7–25.1)		24.7 ± 3.0 (23.4–25.9)				0.380
three months	23.7 ± 2.7 (22.4–24.9)	−0.2 ± 0.7 (−0.6 to 0.1)	24.2 ± 3.0 (23.0–25.4)**a**	−0.4 ± 0.6 (−0.7 to −0.2)			0.517
six months	23.8 ± 2.8 (22.5–25.1)	−0.1 ± 0.9 (−0.5 to 0.3)	24.0 ± 2.8 (22.9–25.1)**b**	−0.7 ± 0.8 (−1.0 to −0.3)**e**			0.808
**Waist−to−hip ratio**					0.530	0.742	0.904
Baseline	0.90 ± 0.05 (0.88–0.92)		0.90 ± 0.05 (0.88–0.92)				0.869
three months	0.90 ± 0.05 (0.88–0.92)	−0.00 ± 0.02 (−0.01 to 0.01)	0.90 ± 0.06 (0.88–0.93)	0.01 ± 0.05 (−0.02 to 0.03)			0.794
six months	0.90 ± 0.05 (0.88–0.93)	0.01 ± 0.03 (−0.01 to 0.02)	0.90 ± 0.05 (0.87–0.92)	0.00 ± 0.02 (−0.01 to 0.01)			0.637
**Body fat rate (%)**					0.350	0.206	0.294
Baseline	32.3 ± 6.0 (29.5–35.0)		34.9 ± 6.7 (32.2–37.6)				0.171
three months	32.5 ± 6.9 (29.3–35.6)	0.2 ± 1.5 (−0.5 to 0.9)	34.0 ± 6.1 (31.5–36.5)	−0.9 ± 3.1 (−2.2 to 0.3)			0.423
six months	32.2 ± 6.6 (29.2–35.2)	−0.1 ± 1.4 (−0.7 to 0.5)	33.8 ± 5.7 (31.6–36.1)	−1.1 ± 3.1 (−2.3 to 0.2)			0.363
**Skeletal muscle index (kg/m2)**					0.508	0.177	0.910
Baseline	14.7 (13.6,16.0)		14.8 (14.0,16.3)				0.966
three months	14.7 (13.7,16.0)	0.0 (−0.4,0.0)	14.5 (13.8,16.2)	−0.1 (−0.4,0.1)			0.949
six months	14.7 (14.0,15.8)	−0.1 (−0.4,0.3)	14.5 (13.9,16.2)	−0.1 (−0.4,0.2)			0.847

Values are presented as means ± SD (95% confidence interval) or median (25%,75%) (if data are not normally distributed). **a:** Significant difference between baseline and three months in the same group; **b:** Significant difference between baseline and six months in the same group; **c:** Significant difference between three and six months in the same group; **d:** The difference value between three months and baseline values was significant between the two groups; **e:** The difference value between three months and six months values was significant between the two groups. Bolded values are significant.

### Physical performance testing results

After 6 months of Yijinjing combined with elastic band exercise, participants’ handgrip strength, gait speed, leg power, eye-closed and single-legged standing and sit-and-reach existed in the interaction of the group and time effect (*p* < 0.05), the time effect were also statistically significant (*p* < 0.05), and the group effect of gait speed was significant different (*p* = 0.022) (Table 4). Reaction time showed no interaction between group and time effect, but the time effect (*p* < 0.001) and intergroup effect (*p* = 0.035) were statistically significant. Compared with baseline, the result at 3 and 6 months in the EG was improved significantly (*p* < 0.05) in term of handgrip strength, gait speed, reaction time, leg power, eye-closed and single-legged standing and sit-and-reach. In the EG, the grip strength, reaction time, leg power, and sit-and-reach between 3 and 6 months showed statistical difference (*p* < 0.05). Gait speed and reaction time between both groups at 3 and 6 months were significantly different (*p* < 0.05), as was leg power at 6 months (*p* < 0.05). In term of grip strength, gait speed, leg power, eye-closed and single-legged standing, and sit-and-reach, the difference values between 3 and/or 6 months and the baseline values were significantly different between the two groups (*p* < 0.05). Compared with baseline, the reaction time of the CG at 6 months was significantly improved (*p* < 0.05), and no other significant changes were observed.

**TABLE 4:** Effect of Yijinjing combined with resistance exercise on physical performance in the study’s participant.

Physical performance	Control (*n* = 21)	Difference from baseline	Exercise (*n* = 26)	Difference from baseline	*P*-value	*P*-value	*P*-value
					Group–time interaction	Time	Group
Handgrip strength (kg)					<**0.01**	**0.003**	0.606
Baseline	22.8 (19.9,31.7)		22.6 (21.4,27.7)				0.864
three months	22.5 (20.0,31.1)	−0.1 (−1.0,0.3)	24.7 (22.4,29.9)**a**	1.4 (0.7,2.1)**d**			0.211
six months	23.6 (19.5,30.1)	−0.9 (−1.6,1.3)	25.5 (23.2,30.8)**bc**	2.1 (1.3,4.1)**e**			0.071
Gait speed (m/s)					**0.003**	<**0.01**	**0.022**
Baseline	1.09 (1.01,1.26)		1.11 (1.01,1.23)				0.923
three months	1.09 (1.05,1.21)	0.00 (−0.12,0.06)	1.27 (1.18,1.34)**a**	0.12 (0.06,0.19)**d**			**0.001**
six months	1.17 (1.10,1.27)	0.03 (−0.08,0.16)	1.32 (1.22,1.40)**b**	0.19 (0.09,0.28)**e**			**0.003**
Reaction time (s)					0.310	<**0.01**	0.035
Baseline	0.460 (0.386,0.517)		0.427 (0.401,0.452)				0.473
three months	0.413 (0.376,0.462)	−0.017 (−0.078,0.005)	0.374 (0.358,0.405)**a**	−0.042 (−0.082, −0.026)			0.025
six months	0.382 (0.366,0.413)**b**	−0.037 (−0.109,−0.009)	0.359 (0.330,0.385)**bc**	−0.066 (−0.119, −0.031)			**0.008**
Leg power (W)					<**0.01**	<**0.01**	0.610
Baseline	248.0 (168.0,301.5)		199.5 (162.0,288.0)				0.435
three months	264.0 (147.5,337.0)	0.0 (−20.0,27.5)	282.5 (223.5,338.5)a	58.5 (13.0,141.8)**d**			0.219
six months	209.0 (155.0,344.0)	−4.0 (−30.0,39.5)	300.5 (263.0,365.0)**bc**	101.5 (43.5,172.3)**e**			**0.026**
Eye-closed and single-legged standing (s)					**0.040**	**0.004**	0.185
Baseline	6.0 (5.0,10.0)		5.0 (3.0,10.0)				0.250
three months	7.0 (5.0,11.0)	0.0 (−1.0,1.5)	9.0 (5.0,16.3)**a**	3.0 (2.0,7.8)**d**			0.175
six months	9.0 (5.0,12.5)	0.0 (−2.5,4.5)	10.0 (6.8,23.0)**b**	6.0 (3.8,12.8)**e**			0.056
Sit-And-Reach (cm)					<**0.01**	<**0.01**	0.882
Baseline	6.0 (3.8,12.3)		3.5 (−3.1,12.0)				0.493
three months	6.0 (3.0,11.8)	0.0 (−0.8,0.5)	7.8 (−0.8,14.0)**a**	1.8 (1.5,2.6)**d**			0.773
six months	5.0 (1.0,11.0)	−0.5 (−2.0,1.0)	9.3 (0.4,14.0)**bc**	3.0 (2.0,4.0)**e**			0.434

Values are presented as means ± SD (95% confidence interval) or median (25%,75%) (if data are not normally distributed). **a:** Significant difference between baseline and three months in the same group; **b:** Significant difference between baseline and six months in the same group; **c:** Significant difference between three and six months in the same group; **d:** The difference value between three months and baseline values was significant between the two groups; **e:** The difference value between three months and six months values was significant between the two groups. Bolded values are significant.

### Blood testing results

**FIGURE 3:**
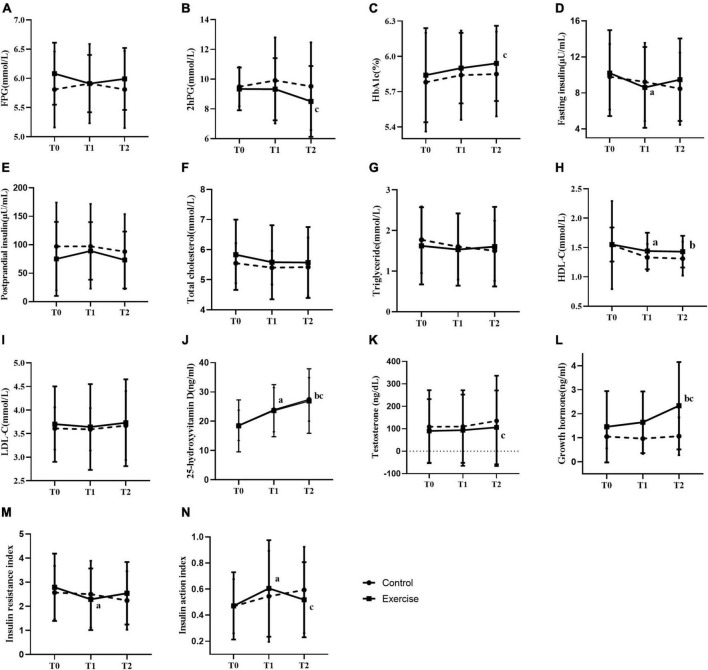
Effect of exercise on glucose and lipid metabolism. **(A–N)** Fasting blood-glucose **(A)**, 2–hour post-meal blood glucose **(B)**, glycated hemoglobin% **(C)**, fasting insulin **(D)**, postprandial serum insulin **(E)**, total cholesterol **(F)**, triglyceride **(G)**, high density lipoprotein cholesterol **(H)**, low density lipoprotein cholesterol **(I)**, 25–hydroxyvitamin D **(J)**, testosterone **(K)**, growth hormone **(L)**, insulin resistance index **(M)**, insulin action index **(N)**. a: significant difference between baseline and the 3rd month in the same group; b: significant difference between baseline and six months in the same group; c: significant difference between 3 months and 6 months in the same group. T0, T1, and T2 represent baseline, 3 months, and 6 months respectively. * represents *p* < 0.05 and ** represents *p* < 0.01.

**FIGURE 4:**
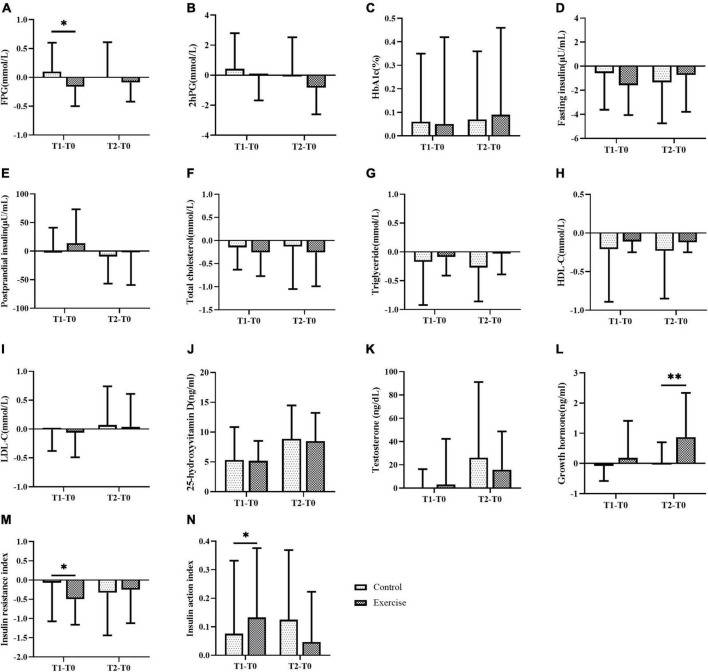
Comparison of difference values between three or six months and baseline in different groups on glucose and lipid metabolism, **(A–N)** fasting blood-glucose, FPG **(A)**, 2–Hour post-meal blood glucose **(B)**, glycated hemoglobin% **(C)**, fasting insulin **(D)**, postprandial serum insulin **(E)**, total cholesterol **(F)**, triglyceride **(G)**, high density lipoprotein cholesterol **(H)**, low density lipoprotein cholesterol **(I)**, 25–hydroxyvitamin D **(J)**, testosterone **(K)**, growth hormone **(L)**, insulin resistance index **(M)**, insulin action index **(N)**. T1–T0 represents the difference value between three months and the baseline, and T2–T0 represents the difference value between six months and the baseline. * represents *p* < 0.05, ** represents *p* < 0.01. The title and note of all tables.

During the exercise intervention, no interaction was found between the group and time, and no intergroup effect (except for growth hormone) was observed on all glucolipid metabolism indicators (Figures 3, 4). FINS, PSI, TC, HDL-C, VD, TTE, GH, and IAI have time effect. Compared with baseline, FPG, 2hPG, FINS, TC, HDL-C, and HOMA2-IR showed a decreasing trend, and the result of FINS and HOMA2-IR at 3 months and the result of HDL-C at 3 and 6 months were significantly decreased in the EG (*p* < 0.05) (Figure 3). Compared with 3 months, the EG’s 2hPG and HbA1c (%) at 6 months were significantly different (*p* < 0.05) (Figures 3B,C). Compared with baseline, GH was significantly higher at 6 months in the EG, and IAI was significantly higher at three months in the EG, with significant differences between 3 and 6 months (Figures 3L,N). Furthermore, in term of FPG, HOMA2-IR, and IAI, the difference values between three months and baseline values were significantly different between the two groups (Figures 4A,M,N), and the difference value of GH between 6 months and baseline values was significant(*p* < 0.01) (Figure 4L). Compared with baseline, the both groups’ VD gradually and significantly increased at 3 and 6 months (*p* < 0.05), and significant difference in VD was observed (Figure 3J). But there wasn’t significant change on TTE levels between the two groups.

## Discussion

To our knowledge, this is the first randomized controlled trial that investigated the effect of Yijinjing combined with elastic band exercise on muscle mass and function in prediabetic patients. First, 6 months of this exercise program significantly reduced body weight, BMI, leg fat mass, gynoid fat mass and total body fat mass, and delayed lean mass loss in patients with PDM. Second, their handgrip strength, gait speed, leg power, balance ability, flexibility and reaction ability were improved significantly. Third, the EG’s FPG, 2hPG, FINS, TC, and HOMA2-IR gradually decreased, and growth hormone was gradually increased.

The incidence of T2DM increases with body weight, body fat, and BMI (21). This study found remarkable reductions in body weight, BMI, leg fat mass, gynoid fat mass, and total body fat mass in the EG compared with those at baseline. This finding was consistent with previous studies showing that aerobics combined with resistance exercise three times a week for 12 weeks can decrease body weight and body fat mass in patients with DM (22). A decrease in lean mass was observed in both groups after 6 months of this program, but the differences were not significant, except for android. Comparisons between baseline and 3 or 6 months showed that the total muscle mass loss in the EG was consistently less than that in the CG, but the difference was not statistically significant. This finding can be attributed to the enhancing effect of Yijinjing combined with resistance exercise on muscle atrophy due to aging and insulin resistance. Colleluori et al. found that aerobic plus resistance exercise could reduce body fat in obese patients and promote muscle protein synthesis, thereby protecting their muscle mass (14). The lack of significant improvement in the muscle mass of the EG can be explained as follows. First, this study only carried out exercise intervention for patients without dietary control or even a reminder to increase their protein intake. Exercise promotes the decomposition rate of muscle protein to exceed the rate of muscle protein synthesis, thus simultaneously reducing fat and muscle mass. Second, the rate of improvement of muscle mass by aerobic combined resistance exercise was less than the rate of muscle atrophy caused by aging and insulin resistance (8). As a result, the muscle mass of the EG decreased, but the reduction was less than that for the CG. Although no significant change in body fat percentage was found in the EG, a gradual decline was noted. Future researchers must attempt to reduce weight and prevent muscle mass loss during weight loss by increasing the duration and intensity of interventions and combining vitamin D and protein supplements (23).

According to a published study in Lancet, grip strength may be a biomarker of aging and can be used to predict all-cause, cardiovascular, and non-cardiovascular mortality; people with high mortality risk from cardiovascular disease have been associated with a low grip strength (24). Gait speed and leg power reflect lower limb muscle mass and strength in patients with PDM and are associated with increased risk of falls, disability, and fractures (25). Eye-closed and single-legged standing, reaction time, and sit-and-reach represent balance ability, reaction ability and flexibility, respectively. These physical qualities are important in the daily activities of middle-aged and elderly people. This study found that the handgrip strength, gait speed, reaction time, leg power, eye-closed and single-legged standing, and sit-and-reach were significantly improved in the EG compared with those in the CG (*p* < 0.05). This finding was consistent with Villareal et al., who reported that 6 months of aerobic combined resistance exercise significantly improved lower limb strength (18%), gait speed (14%), balance (13%), and SF-36 physical-component (24%) (23). In addition, several studies reported that the Yijinjing could improve the muscle contraction (26), muscle strength (27), and balance function (28) of patients with Skeletal muscle loss. This also reflects indirectly the beneficial effects of the combined exercise on muscle mass and function of PDM patients.

For the proposed exercise program, elastic band exercises such as straight-arm chest expansion and forward lunge biceps bending were specially designed to stimulate the upper limb muscle groups and increase the arm muscle strength to prevent the fall and post-fall injury accidents of elderly patients with PDM in the early stage. Yijinjing can effectively enhance skeletal muscle function, improve lower limb muscle strength, balance and flexibility, and increase walking stability in patients with sarcopenia, thereby improving their quality of life (29, 30). Yijinjing stretch, twist, turn, swinging, ups and downs, constantly pull around the hip joint and each part of the total body muscle, fascia, ligaments and connective tissue, promoting blood circulation to each part of the total body soft tissue to restore joint synovial fluid, activate the stiff muscles and joints, so as to increase the range of activities and muscle power (31). Yijinjing and elastic band exercise is composed of many squat exercises that require patients to repeatedly accelerate and decelerate, constantly stimulate the motor nervous system, and activate the hip muscles to increase the muscle strength of lower limbs and lower limb explosive force. Yijinjing combined with elastic band exercise can exercise the muscle strength of all parts of the patient’s body, help patients in standing, squatting, lunging and other positions to perceive the force of all parts of the body, so as to improve the patient’s balance ability (28). The improvement of the patient’s physical quality in all aspects of the body makes the patient’s gait more stable and faster in daily walking. Therefore, Yijinjing combined with elastic band exercise may effectively improve the physical performance of patients with PDM.

Abnormal glucose and lipid metabolism in patients with PDM is associated with diabetes complications, disability and increased mortality, however, the effect of exercise on the prevention and treatment of diabetes has been widely recognized. Traditional Chinese Exercises (TCEs) regard TCM as the theoretical guide, including Taijiquan, Wuqinxi, Baduanjin, Liuzijue, Yijinjing, and so on. TCEs are a non-medicine therapy for DM and PDM, and can strengthen the body, and prevent and cure diseases (32). In recent years, studies have found that long-term Tai Chi exercises can decrease blood glucose and HbA1c levels (33); Baduanjin can reduce glucose metabolism disorders and stabilize blood sugar and HbA1c levels (34); Wuqinxi can improve blood pressure, HDL-C and LDL-C, alleviate the cardiovascular disease risk factors associated with metabolic syndrome (35). Yijinjing can improve liver and spleen function effectively in patients with T2DM to stabilize blood sugar levels (36). At present, a majority of research in this field utilizes randomised controlled trials (RCTs) to support the clinical efficacy of a single TCE on PDM or DM, and there is no RCT comparing the clinical efficacy between Yijinjing and other TCEs for the diseases (37). A network meta-analysis of RCTs shown that Wuqinxi may be the best TCE treatment for dyslipidemia in the middle-aged and elderly (38). However, there are several limitations of the meta-analysis. Firstly, TCEs were discussed only from the perspective of dyslipidemia, without comparing the effects of TCEs on blood glucose, muscle function and muscle mass. Secondly, only four Chinese articles on Yijinjing were included, and Yijinjing was not compared with other TCEs; Thirdly, different RCT studies had different intervention programs and study populations, and no article directly compared the intervention effects of the six TCEs on the blood control. Thus, the best one of TCEs for PDM and DM needs to be further explored.

In this study, FPG, 2hPG, FINS, TC, and HOMA-IR in the EG showed no significant changes, compared with the CG, but the overall trend was continuously decreasing. Previous studies found that the FPG, 2hPG, and TC of T2DM patients did not change significantly before and after 12 months of resistance exercise or aerobic exercise, but showed an overall downward trend, which was similar to our results (39). This may be the effect of Yijinjing combined with elastic band exercise to improve blood glucose control in prediabetic patients. Tomas et al. found that 12 weeks of aerobic combined resistance exercise significantly improved muscle strength, blood glucose control, and health-related quality of life in patients with T2DM (40). HOMA-IR was significantly reduced in the EG at 3 months compared with baseline, but not at 6 months. It may be because skeletal muscle increased blood sugar and other energy consumption in the first 3 months to adapt to exercise consumption, and the participants developed exercise habits in the last 3 months. After gradually adapting to this exercise mode, the effect of exercise on blood sugar control of patients is weakened. Our study found that HbA1c levels increased gradually in both groups, with similar increases, and there was a significant difference in HbA1c levels in the EG at 3 and 6 months. However, many studies have reported (41) that aerobic combined with resistance exercise can reduce HbA1c level in patients with T2DM, which is contrary to the current findings. The present participants were patients with PDM, and their baseline HbA1c% was lower than that of patients with T2DM. Hence, exercise had a small effect on HbA1c%, and the influence of patients’ daily diet on exercise efficiency could not be excluded. Some studies (42) showed that LDL-C significantly decreased after aerobic and resistance exercise; however, no significant change in LDL-C was recorded in the present work possibly due to differences in exercise time and intensity. The reduction in HDL-C in the EG was surprising because most studies have reported no change or improvement (43). Jorge et al. investigated the effects of aerobic, resistance, and combined exercises on metabolic control in patients with T2DM and found that HDL-C showed no significant change in the combined group but was significantly reduced in the control, aerobic, and resistance groups (44). At present, the mechanism of exercise-induced changes in HDL-C of patients with PDM and T2DM remains to be further explored. Compared with those of the CG, the FPG, HOMA2-IR, and IAI of the EG were significantly different between the 3rd month and baseline.

Insulin resistance and hormonal changes interact and worsen each other. Insulin resistance is accompanied by the downregulation of TTE and GH, which are involved in muscle anabolism and catabolism. Reduced TTE and GH levels can weaken muscle mass and function (45), leading to sarcopenic obesity. The results indicate that Yijinjing combined with elastic band exercise can effectively promote the secretion of GH in patients with PDM but has minimal effect on TTE levels. Decreased outdoor physical activity and insufficient UV exposure can inhibit VD production (46). VD deficiency accelerates muscle atrophy (47), and its supplementation positively affect muscle strength, athletic performance, and fall prevention in older adults. In the present study, VD levels were significantly higher at 3 and 6 months compared with that at baseline in all participants. Given that the experiment was carried out in spring and summer, sun exposure was encouraged among the patients and might have promoted their VD production. Therefore, we hypothesize that Yijinjing combined with elastic band exercise had minimal effect on VD in patients with PDM, far less than the promotion from sun exposure.

This study had several limitations. First, the participants’ diet and daily activities failed to be controlled and that possibly affected the effect of exercise. Future trial interventions may combine exercise with protein supplements, and also monitoring of participants’ daily activities. In addition, our sample size was not large enough, and the majority of participants were educated Asian females which limited wider generalizability of the results. Future research should expand the sample size and include participants of different gender, age groups and race.

In conclusion, Yijinjing combined with elastic band exercise can reduce the body weight, BMI, and fat content of middle-aged and elderly patients with prediabetes, improve their muscle function and growth hormone secretion, and delay muscle mass loss and diabetes development.

## Data availability statement

The original contributions presented in this study are included in the article/supplementary material, further inquiries can be directed to the corresponding author.

## Ethics statement

The studies involving human participants were reviewed and approved by the Ethics Committee of Scientific Research of Shanghai University of Sport (Ethics batch number: 102772021RT066). The patients/participants provided their written informed consent to participate in this study.

## Author contributions

YDH and XYL designed the study and analyzed the data. JHH, QG, and SJW diagnosed diseases and recruited participants. YDH, YWC, JYL, and SSW instructed participants to exercise and acquired and collected the data. YDH drafted and revised the manuscript. XYL critically reviewed and revised the manuscript. RW provided the support of funding. All authors contributed to this article and approved the submitted version.
